# Forecasting alien species establishment and source regions: Quantitative assessment of potential ant invasions in Japan

**DOI:** 10.1002/eap.70071

**Published:** 2025-07-14

**Authors:** Yazmín Zurápiti, Jamie M. Kass, Benoit Guénard, Evan P. Economo

**Affiliations:** ^1^ Biodiversity and Biocomplexity Unit Okinawa Institute of Science and Technology Onna Okinawa Japan; ^2^ Macroecology Laboratory Graduate School of Life Sciences, Tohoku University Sendai Miyagi Japan; ^3^ School of Biological Sciences The University of Hong Kong Pokfulam Hong Kong

**Keywords:** invasion process, model transfer, prediction of alien establishment, risk management, species distribution model, transfer evaluation

## Abstract

Due to the costs and difficulties of mitigating past biological invasions, there is a critical need for improved predictions of establishment risk for alien species and their source regions to guide the deployment of preventive measures. Here, focusing on a global pool of ant species known to be spread by humans, we develop a computational workflow to predict threats for a country or region of interest. Specifically, the workflow (1) predicts which alien species are most likely to be established based on climatic suitability with species distribution models, (2) clusters areas threatened by similar assemblages of alien species, and (3) identifies global regions that can act as important sources for these species. We apply this workflow to estimate which ants with human‐assisted invasion histories around the globe may establish in Japan, an island country with broad climatic and topographic diversity. To reduce forecast uncertainty, we exclude models that we assess to result in dubious transfers based on evaluations of species already established in Japan and avoid making model extrapolations. To better account for the full invasion process, we also estimate introduction risk and spread within Japan and integrate these with our establishment risk and potential sources estimates. Our results indicate that all prefectures of Japan have potential risks of new alien ant establishments, though lower latitudes and small archipelagoes have the highest predicted vulnerability. When combined with the likelihood of spread, we expect shifts in vulnerability toward highly populated areas and in proximity to international ports. Interestingly, the source regions with the most alien species presenting establishment threats are in southern Europe and the subtropical Americas rather than in Asia, in part because many Asian species have already been introduced to Japan. When considering introduction risk based on global trade patterns, the United States was most likely to be a source of future introductions. We discuss the implications of these results for global management policies and cargo surveillance. The workflow described here can be deployed worldwide for different taxa to predict the establishment potential of alien invasions and their sources, and also to design more practical and efficient preventive strategies.

## 要約

すでに侵入してしまった外来生物による被害の軽減には多大な費用と困難が伴うため、外来種の定着リスクおよびその侵入源をより正確に予測し、予防措置の展開を指導することが緊急の課題となっている。ここでは、人為的に分布を拡大することが知られているアリ類の世界的なプールに焦点を当て、対象となる国または地域に対する脅威を予測する計算ワークフローを開発した。具体的には、このワークフローは (1)気候適性に基づく種分布モデルによる定着可能性が高い外来種の予測、(2) 類似した外来種群に脅かされる地域のクラスター化 (3)これらの種の重要な供給源となり得る地域を特定の3要素により構成される。本研究では、このワークフローを適用して、世界中で人為的に侵入が記録されているアリ類のうち、気候や地形が多様な島国である日本に定着する可能性がある種を推定した。予測の不確実性を低減するため、すでに日本に定着している種の評価をもとに、疑わしい推定がされるモデルを除外し、モデルの外挿を避けた。また、侵入プロセス全体をより正確に考慮するため、日本国内での導入リスクや拡散の可能性も推定し、これらを定着リスクおよび潜在的な供給源の推定結果と統合した。その結果、日本のすべての都道府県に新たな外来種のアリ類が定着するリスクがあることが示され、低緯度地域や小さな群島において最も脆弱性が高いと予測された。さらに、人為的分散の可能性を考慮に入れると、脆弱性は人口密集地や国際港に近い地域にシフトすることが予想される。興味深いことに、最も多くの外来種が定着の脅威を示す侵入源は、アジアではなく南ヨーロッパと亜熱帯アメリカにあることが判明した。これは、多くのアジア産種がすでに日本に侵入しているためと考えられる。世界の貿易パターンに基づく導入リスクを考慮した場合、将来的な導入の供給源として最も可能性が高いのはアメリカ合衆国だった。これらの結果を踏まえ、世界的な管理政策や貨物監視の方法について考察する。ここで示したワークフローは、外来侵入の定着可能性とその供給源を予測するために、世界中の様々な分類群に対して展開でき、より実用的かつ効率の高い予防戦略の設計にも寄与することが期待される。.

## INTRODUCTION

Over 16,000 alien species have established outside their native ranges (Seebens et al., 2018) and more than 2000 are considered invasive alien species (only 0.1% of global biodiversity) that cause severe damage to ecosystems and people (Larsen et al., 2017; May, 1988; Pimentel et al., 2001). Alien species are predicted to continue spreading and accumulate at continental scales, and the estimated pool of species that might become new threats increases with expanding trading networks (Seebens et al., 2018, 2021). The removal of dispersal barriers through increased human transport via globalization (Bertelsmeier et al., 2017; Bonnamour et al., 2021; Costello et al., 2007; Huenneke et al., 1988) thus has severe consequences such as habitat loss, damage to agriculture and infrastructure, water contamination, and spread of diseases (Diagne et al., 2021; Pimentel et al., 2001; Woodworth et al., 2005). Despite their low proportion of total biodiversity, the recorded costs of the negative impacts of invasive alien species exceed US$20 billion annually, with an estimated cumulative total up to 2017 of nearly US$1.3 trillion (Bradshaw et al., 2016; Diagne et al., 2020, 2021) and increasing globally every year (Diagne et al., 2021).

Significant efforts have been made to understand and manage the global spread and ecological consequences of specific invasive alien species with well‐documented harmful effects. For example, the drifting dinoflagellate *Ceratium furcoides* reduces water quality to toxic levels and has become a major concern in invaded countries, motivating research on their distributional spread and niche shifts (Macêdo et al., 2021). To combat the spread of human diseases, the ecology, native distribution, and spread of vector taxa such as mosquitoes have a long history of investigation (Alaniz et al., 2019; Nelson, 1986). However, alien species with no direct impact on humans such as nonvenomous animals (Soto et al., 2022) and those that are only possible emerging threats (Fournier et al., 2019; Seebens et al., 2018) can induce potentially severe ecological and economical damage that is difficult to predict. Therefore, to inform control measures, there is a pressing need for better predictions of invasion risk, especially for species of potential concern that may not receive much research attention (Cuthbert et al., 2022; Leung et al., 2002).

Developing preventive programs against new invasions is generally more cost‐efficient than mitigation (Cuthbert et al., 2022; Leung et al., 2002), but this requires not only identifying the likelihood of establishment of potential alien species but also determining their source regions; these are all potential areas they could originate from (Baker, 2017; Wong et al., 2023; Xu et al., 2022). Although many studies have traditionally assumed that species' native ranges serve as the main source region (Richmond et al., 2010), modern high levels of human movement and trade favor new introductions from any region where an alien species is already established. This bridgehead effect has been reported for vertebrates (Louppe et al., 2020) and insects (Lombaert et al., 2010; Xu et al., 2022), and it could involve the potential new introductions of multiple species at once (Moniuszko et al., 2018). Thus, forecasting invasion risk and global source regions for whole communities and higher taxonomic groups should be valuable for nationwide and regional planning.

To date, much research has focused on forecasting invasion potential with species distribution models (SDMs; Srivastava et al., 2019). These models use species' occurrence data and environmental predictor variables to estimate niche relationships between the species and the environment and predict potential geographic distributions. Importantly, they can be employed to make predictions of range in areas without current occurrence records, which is termed “model transfer” (Escobar et al., 2014; Jiménez‐Valverde et al., 2011). Therefore, SDMs can be used to predict species' potential distributions should they be successfully established in a new region. Such models have proven to be valuable tools in predicting the spread of alien species across diverse regions (Jiménez‐Valverde et al., 2011; Srivastava et al., 2019). But several important and unresolved issues persist for SDM predictions of species invasions. One is how to evaluate the accuracy and ecological realism of model transfers, which can necessitate considerable extrapolation when we lack available data for comparisons (see Sutton & Martin, 2022). Another is how to identify potential source regions for invasions when investigating multiple species simultaneously, such as for predictions across the global species pool or key taxonomic groups.

While invasive vascular plants and vertebrates have mostly originated from intentional introductions (Huenneke et al., 1988; Saul et al., 2017), most invertebrates, fungi, and microorganisms have been introduced unintentionally (Saul et al., 2017; Suarez et al., 2005). For instance, the ants (Hymenoptera: Formicidae) include some of the most damaging invasive alien species in the world (Angulo et al., 2022). Two‐thirds of the ant species that frequently pass unnoticed at border control are litter‐ and soil‐dwelling and can be introduced as individual queens or very small colonies in large volumes of cargo where current screening methods remain blind to their presence due to their small size and crypticity (McGlynn, 1999; Wong et al., 2023). With over 300 species established outside their native ranges (Wong et al., 2023), five species listed in the 100 of the world's worst invasive alien species (Lowe et al., 2000), and a cumulative documented total cost of 10 billion US dollars from 1930 to 2017 (Angulo et al., 2022), ants are among the arthropod taxa of greatest concern. Over the past decade, there has been a strong effort to compile biodiversity information from disparate sources with varying accessibility into consolidated databases like the Global Biodiversity Information Facility (GBIF, 2023) or Biological Library (BioLib, 2023). In particular, the Global Ant Biodiversity Informatics (GABI) database has consolidated and curated comprehensive distributional data, including alien (exotic) records, on described ant species (over 16,000 species and subspecies) from diverse sources (Guénard et al., 2017; Wong et al., 2023).

Island‐like systems are particularly susceptible to ecological and socioeconomic devastation by human‐mediated biological introductions (Reaser et al., 2007) and there is a disproportionate number of established alien species on isolated islands (Liu et al., 2023; Moser et al., 2018). Ant invasions throughout islands in the Pacific Ocean have become a major concern, as lack of awareness and preparedness have unintentionally facilitated some of the most costly invasions in the world (Xu et al., 2022). Most countries in the region have implemented mitigation measures only after the detection of alien species (Xu et al., 2022). For example, Japan has incurred an estimated total cost of 728 million USD from 1965 to 2017 due to biological invasions (Watari et al., 2021), and management of smaller remote archipelagoes has been more expensive than that for mainland regions despite their extensive latitudinal span and wide range of biomes (Watari et al., 2021). Moreover, despite being extensively connected through transcontinental trade (Hu et al., 2020; Mou et al., 2018), current containment border controls only target specific ant species (mainly *Solenopsis genus*, *Linepithema humile*, *Lepisiota frauenfeldi*, and *Wasmannia auropunctata*) while other species are practically ignored, which reveals a general lack of preparedness (Xu et al., 2022).

In this study, we develop a computational workflow to make predictions of establishment risk in a country or region of interest by identifying the threat of alien species and their source regions. We demonstrate its applicability by assessing the potential of alien ants from a global pool of candidates to establish in Japan, identify which species threaten specific areas in Japan, and infer the geographic regions that contain multiple threatening species. To do this, we use SDMs trained on these species' inhabited ranges to predict potential ranges for species with no history of invasions in Japan. To avoid ecologically unrealistic results, we exclude predictions with poor model transfer expectations, which we infer based on the performance of transfers for alien species already established in the country, and additionally remove areas of high model extrapolation to reduce uncertainty. The ecological analyses presented here extend previous efforts to understand the spread of invasive ant species (Fournier et al., 2019; Suarez et al., 2005) by combining curated range predictions to make risk assessment maps of potential new establishments and global source regions. Additionally, we estimated introduction risk based on the volume and country of departure for the incoming cargo to Japan and combined it with our estimates of establishment risk to incorporate the effect of trade on the ecological predictions. We end by discussing how this approach could facilitate the implementation of practical border control measures by creating prevention protocols based on trade networks (Costello et al., 2007; Xu et al., 2022) rather than on individual species that might be difficult to track and identify by nonexperts (Wong et al., 2023).

## METHODS

**FIGURE 1:**
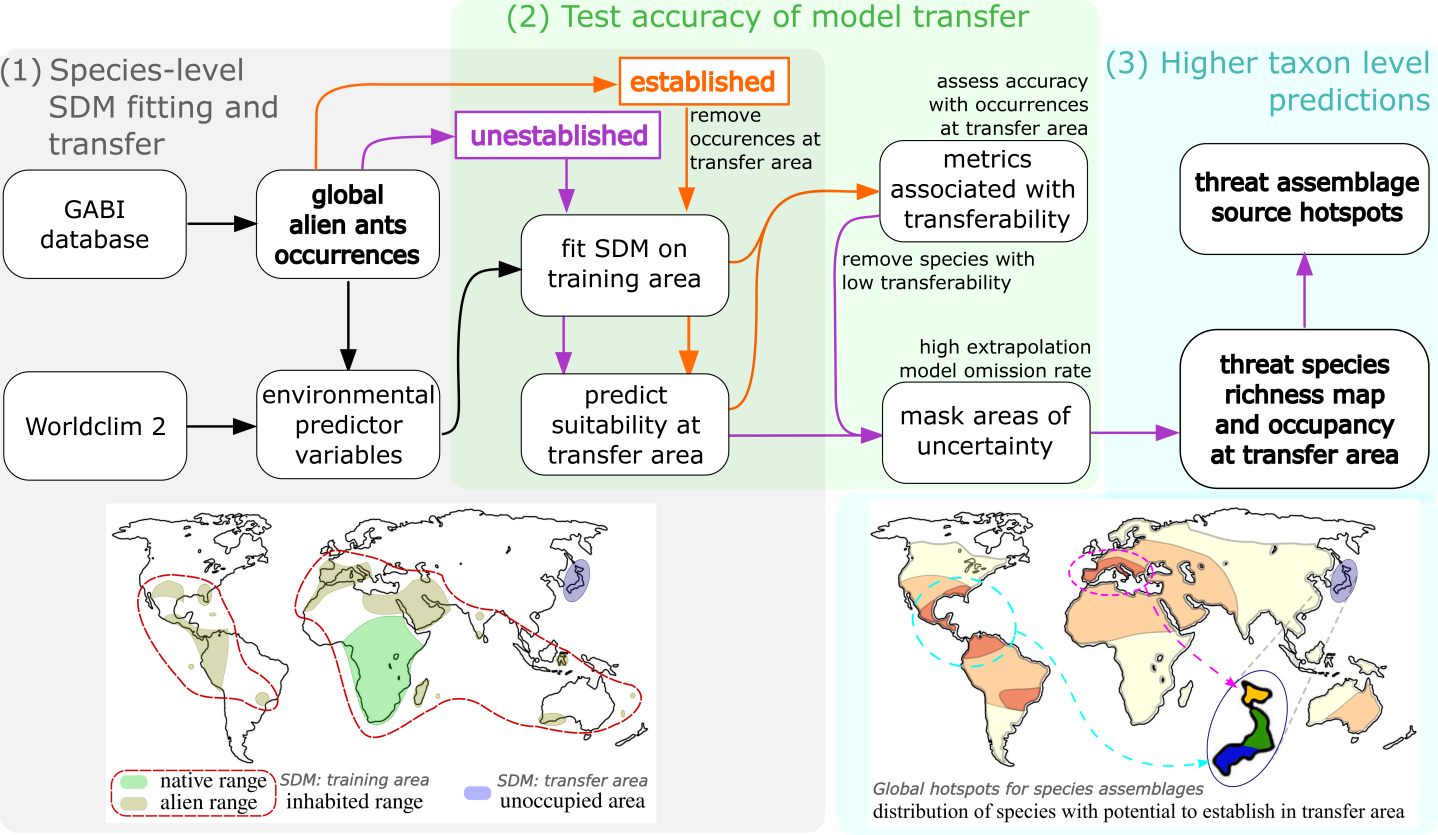
Analysis workflow to predict potential alien establishment and sources. The workflow has three main components. Step 1 corresponds to the fitting of a species distribution model (SDM) and transferring it to the area of interest. Species are split into “established” (those that already have occurrence points in the transfer area; for this study, Japan) and “unestablished” (those for which we are interested in predicting potential establishment). To test the accuracy of the transfer in step 2, models for established species are fitted only with the occurrence points outside the transfer area, and the points inside the transfer area are used as independent test data. In step 2, the established species' transfers are used to derive which input data and model‐training metrics (and values therein) are associated with low transferability, or those that were indicative of dubious transfers. The derived transferability evaluation metrics are used to exclude unestablished species with transferability metric values that lie in the range of dubious transfers to prevent including poor transfers in the predictions. Finally, in step 3 risk predictions are produced by combining the curated unestablished species predictions to estimate potential alien richness, define species threat assemblages, and find the regions of the world with similar composition (source hotspots).

For terms referring to the invasion process, we used the terminology proposed by Blackburn et al. (2011): briefly, we refer to “prevention” as management before transportation of an alien species to a new place, “containment” as responses to prevent the introduction of an alien species to a region, and “mitigation” as all measures taken to prevent further spread of an already introduced alien species, including eradication attempts. Then, we define the “inhabited range” as anywhere where an alien species is already established outdoors (both native and alien ranges; Figure 1), with the assumption that the species could be freely transported to unoccupied areas via human intervention. The inhabited range defines the training area of the SDM, and the transfer area is the region or country of interest, an unoccupied area (outside the training area) that defines the extent of model transfer (Figure 1). In our study, Japan is the transfer area, and we want to assess the risk of establishment and potential source regions for ants that have alien records elsewhere in the world. In the following, we refer to a species not native to Japan that has already established populations in the country as “established,” and to a species that does not have occurrence records in the country as “unestablished” (Figure 1). All analyses were done using R version 4.2.1 (2022‐06‐23; R Core Team, 2022) on an x86_64‐pc‐linux‐gnu (64‐bit) platform running CentOS Linux 8. The workflow's code is available on Zenodo in Zurápiti et al. (2025).

### Biodiversity data

We downloaded species' occurrence data from GABI database version 11 (downloaded on May 2022; Guénard et al., 2017). The GABI database compiles ant species distributional records from 8811 publications over multiple years and in 24 languages, including records from online databases, museums, and personal collections of taxonomists. From these resources, available information on current taxonomy and geographic validity has been curated and adapted to a standard format (Guénard et al., 2017). We used only records with high taxonomic certainty and attributed as “valid” in the database, and we excluded records labeled as “need verification” and “dubious” (Guénard et al., 2017). For our models, to avoid including occurrence records not influenced by climate variables, we limited our download to records with regional status “native” or “exotic” and excluded “transport only” and “indoor introduced” records (Guénard et al., 2017; Wong et al., 2023). Exotic records correspond to specimens collected outdoors and not specifically associated with an indoor nest, thus reflecting established populations under the direct influence of local climate (Guénard et al., 2017; Wong et al., 2023). We used a version of the data that have been cleaned and geocoded to produce coordinates for records that lacked them originally (Kass et al., 2022). We spatially thinned the species' occurrence records for modeling to reduce biases associated with spatial clustering following a previously published protocol (Kass et al., 2022). To match the resolution of the occurrence points with the environmental raster data described below (Moudrý et al., 2023), we used a distance of 20 km to thin the occurrence records. In similar studies, between 14 and 25 records have been shown to produce robust models, depending on the size of the species' range (van Proosdij et al., 2016); therefore, we only included species with at least 20 cleaned global records (from the candidate pool of alien species) in our workflow. The compiled ant occurrence data are available on Zenodo in Zurápiti et al. (2025). For analysis, the candidate pool of alien species was further split into two groups: (1) those already established in Japan and (2) those yet unestablished. Each group followed different workflows (Figure 1). For the established species, we excluded alien occurrence records in Japan from the training data prior to modeling and then used them to evaluate model accuracy in order to assess transfer accuracy and ecological realism for the unestablished species (Figure 1).

### Environmental variables

For environmental predictor variables, we used the bioclimatic rasters from Worldclim 2 (Fick & Hijmans, 2017) at 2.5 arc minute resolution (~5 km), masked to remove water bodies (Kass et al., 2022). We removed variables representing interactions between temperature and precipitation (bio 8, bio 9, bio 18, bio 19) because they presented discontinuities within the training areas of several species (Booth, 2022). We then reduced this set of variables to improve the realism of the model predictions (Sheppard, 2013) and avoid overfitting (Jiménez‐Valverde et al., 2011; Phillips et al., 2017). To do this, we ran a principal components analysis (PCA) on the bioclimatic values of the combined occurrence records and selected those that both contributed the most to the principal components and maintained pairwise Pearson correlation coefficients below 0.85. The final variable set was composed of the following six variables: temperature annual range (bio 7), mean temperature of warmest quarter (bio 10), mean temperature of coldest quarter (bio 11), precipitation seasonality (bio 15), precipitation of wettest month (bio 13), and precipitation of driest month (bio 14). We defined the transfer area by masking the variable rasters to the vector shape of Japan from Natural Earth (Natural Earth, 2022) obtained through the R package *rnaturalearth* (Massicotte & South, 2023). While previous research has shown that species interactions and habitat data can improve the accuracy of SDMs (Wisz et al., 2013), we decided to focus on climatic predictors for this study. First, as the important species interactions across the ranges of most ant species are unknown, it is not feasible to determine which are most relevant to use as predictor variables. Furthermore, most alien ants have generalist associations with large taxonomic groups relating to predation and nesting (Holway et al., 2002; Nathan et al., 2023). Second, as it is not advised to pair historical occurrence records with predictor variables describing contemporary conditions (Bracken et al., 2022), we employed climatic data representing long‐term averages and extremes, and not habitat variables that represent snapshots of current land cover or land use.

### Species distribution modeling

We fitted presence‐background SDMs on the training area of each species (step 1 of the workflow; Figure 1) using the algorithm *Maxent 3.4.4* (Phillips et al., 2017). We chose to use this single algorithm instead of a model ensemble as Maxent features a range of flexible model fits, consistently ranks as high‐performing across a range of large datasets (Valavi et al., 2022), and has even been shown to outperform ensembles for the purposes of transferring models to new conditions (Crimmins et al., 2013; Kaky et al., 2020; Sutton & Martin, 2022; Zhu & Peterson, 2017). We delineated an alpha hull around the occurrence records of each species to represent its study extent using the R package *alphahull* (Pateiro‐López & Rodríguez‐Casal, 2010) with an alpha value of 15. Then, randomly sampled 10,000 background points within this shape using a bias layer (Kass et al., 2022; Phillips et al., 2009). This bias layer represents the sampling density of all GABI occurrence records to account for known sampling biases in the GABI data (Kass et al., 2022). We used the R package *ENMeval 2.0.4* (Kass et al., 2021) to test the performance of a highly flexible model (allowed feature classes: linear, quadratic, and hinge) and a range of complexity penalization strengths (regularization multipliers: 2, 3, 4, and 5, with 5 being the strongest) that either allowed for high model complexity or enforced simplicity. All models used default clamping to restrict predictions to the predictor variable ranges of the training data and avoid free extrapolation.

To evaluate model predictive performance to new conditions (transferability), we applied spatial cross‐validation in *ENMeval* using the “block” partition option, which separates the data spatially into four evenly sized groups. We selected models following the same sequential criteria on performance metrics as Kass et al. (2022): (1) select models with at least one nonzero coefficient, (2) remove models that perform worse than random via the Continuous Boyce Index calculated on the full dataset (CBI; Hirzel et al., 2006), (3) select models with the lowest 10‐percentile omission rate (OR10), and (4) if more than one model remains, select the model with the highest validation value for the Area Under the Curve of the receiver operating characteristic (AUC; Fielding & Bell, 1997). For (2), we removed models with a training CBI below zero or NA; positive values for CBI indicate that the model's predictions are consistent with the distribution of occurrence data (Kass et al., 2022). For (4) we used AUC only to break ties in omission rate and not to evaluate absolute model performance (see issues raised in Lobo et al., 2008). Finally, we made range projections to Japan for the selected model for each species using the predict function in the *dismo* package (Hijmans et al., 2022).

### Assessment of accuracy and realism

As unestablished species have never invaded Japan and lack occurrences there, we cannot evaluate the performance of their model transfers to Japan, and thus we inferred the transferability of SDMs for unestablished species by determining the accuracy and realism for SDMs for established species and developing transferability assessment criteria based on the latter (step 2 of the workflow; Figure 1). To implement this, we first evaluated the ability of established species SDMs to predict alien records in Japan by categorizing the realism of the predictions based on the species' known biology (Appendix S1: Section S1), then measuring the agreement between the model transfer and the independent test data (occurrence points in Japan) by calculating model evaluation metrics (Pearson correlation [cor], AUC, and CBI; Appendix S1: Sections S2 and S3). We followed by examining the relationships between the transfer accuracy metrics and characteristics of the input data and model‐training metrics (Appendix S1: Section S4). Based on those evaluations, we selected the input data and model‐validation metrics (i.e., from spatial cross‐validation) that best explained transfer realism, which we categorized into “expected” or “dubious” suitability patterns. We then developed criteria based on values of evaluation metrics to infer poor transfers in general (see Appendix S1 for details). Finally, we used the developed criteria to identify which unestablished species' models are expected to result in unrealistic predictions for the transfer area and filtered those species out. The remaining unestablished species are expected to have reasonably accurate transfers, and only this curated set was used further in the workflow (Figure 1). For the curated set, we masked areas of transfer uncertainty from their predictions in two ways: (1) we removed areas where predicted suitability values fell below the value corresponding to the lowest 10 percentile (used to calculate the OR10 metric employed in model tuning) and (2) we calculated a multivariate environmental similarity surface (MESS; Elith et al., 2010) between the training and transfer areas using the *dismo* package (Hijmans et al., 2022) and removed areas with negative values, which correspond to areas associated with extrapolation to bioclimatic values outside the training range of the SDM. After these filtering and curating steps, if an unestablished species' prediction has a suitability value (which will be positive and above their corresponding OR10) in any part of the country, it is considered to represent a threat of establishment, thus we refer to them as “threat species.”

### Prediction of the risk of establishment and potential global sources

We calculated the risk of establishment for Japan as the estimated richness of the threat species (step 3 of the workflow; Figure 1), which we obtained by summing their continuous suitability projections (Calabrese et al., 2014). Additionally, we estimated administration‐level species occupancy by calculating the average suitability of each species within each Japanese prefecture. Then, we ran a hierarchical cluster analysis (Euclidean distance and clustering method ward.D2 with the *stats* R package; R Core Team, 2022) to group the prefectures into broader areas (clusters) with similar predicted ant assemblages. We estimated the global potential source areas for the threat assemblage of each cluster from the SDM predictions in the species' inhabited ranges (outside Japan). To do so, we first removed predicted areas below the 10 percentile suitability value as in Kass et al. (2022) and then summed the continuous suitability predictions (Calabrese et al., 2014) weighted by their mean suitability in the cluster. Therefore, source region values represent the weighted richness of unestablished species with the potential to be established in Japan, emphasizing those species with higher predicted suitability in the cluster. Finally, from the resulting source global maps, we identified potential hotspots that represent areas in the world with species compositions similar to the ones predicted to have high establishment risk in each defined cluster.

### Analysis of introduction risk and human dispersal

The main objective of the analysis was to predict the potential of alien species to establish in different regions of a country from a climatic niche perspective (Figure 1). This gives an indication, for example, that cargo from one area may be more likely to contain species with high establishment threat. However, this “per unit cargo” does not incorporate the potential for transport based on realized trade patterns. For example, while source country A may hold more threatening species than source country B, if source country B has higher volumes of trade with the country of interest, it may be more likely to be a source of alien introductions. To make predictions that incorporate both risks, of establishment and introduction, we included an analysis of introduction risk for Japan that clarifies how trade networks modify the ecological predictions. To estimate introduction risk, we used a reasoning similar to (Early et al., 2016) that considers both international trade and human‐aided dispersal after introduction. Since most ant species that pass border controls are associated with large volumes of soil cargo (Wong et al., 2023), we treated seaports as introduction epicenters and considered that newly introduced species could reach their final destination aided by human transportation, which would likely follow human population density patterns (Early et al., 2016).

To represent the introduction risk based on international trade we obtained, for all countries in the world, the mean ocean cargo value in US dollars sent to Japan from 2015 to 2023 (UN Comtrade Database, 2025), and rescaled between 0 and 1 (details in Appendix S1: Section S5, Figure S6a). Then, we multiplied the countries' mean trade value by our previously estimated global weighted richness of threat species to Japan (estimated source regions). To incorporate the potential of spread within the country, we obtained the location and size of seaports from the Ministry of Land, Infrastructure Transport and Tourism websites (National Land Numerical Information Download Site, 2025; Port‐Related Statistical Data, 2025) and interpolated the distance of any point in Japan to the largest and closest port. Then, we multiplied that distance by the logarithm of the population density (“Heisei 22 Census” corresponding to the year 2014; National Land Numerical Information Download Site, 2025). This combines both the likelihood of introduction to a port given the port's size and the dispersal due to human activities of any new alien species. We rescaled these values to be between 0 and 1, which we then multiplied to the establishment risk values (estimated richness of unestablished species; see Appendix S1: Section S5 and Figure S6b for details). The compiled trade and population density data are available on Zenodo in Zurápiti et al. (2025).

## RESULTS

Of the 14,358 ant species in the GABI database, 267 species had at least one exotic record and 234 species had 20 or more global records after spatial thinning. Because we are focusing on country‐level predictions, we excluded the 16 species native to Japan from the analysis. Of the remaining 218 ants with exotic records, 33 were already established in Japan (15,031 total records, of which 14,423 were outside Japan), and 185 were unestablished (49,599 total records). SDMs for all species considered had good performance on training data (training AUC and CBI) and varying performance on withheld data via spatial cross‐validation (validation AUC and CBI; Appendix S1: Section S3).

### Assessment of accuracy and realism for SDM transfers of established species

From the 33 established species, 24 species had transfers with suitability patterns that matched the occurrence data in Japan (“expected” pattern; Appendix S1: Section S1, Figure S1), and 9 species had “dubious” transfers (Appendix S1: Section S1, Figures S2 and S3). Of those with dubious transfers, four species had predictions “opposite” to their known occurrences in the country (*Cardiocondyla itsukii*, *Cardiocondyla kagutsuchi*, *Tetramorium kraepelini*, *Tetramorium smithi*; Appendix S1: Figure S2), and five species had “extreme” predictions, which were largely overpredicted or geographically bimodal, but their known occurrences do not match such patterns (*Hypoponera ragusai*, *Nylanderia amia*, *Ooceraea biroi*, *Pheidole parva*, *Tetraponera allaborans*; Appendix S1: Figure S3).

**FIGURE 2:**
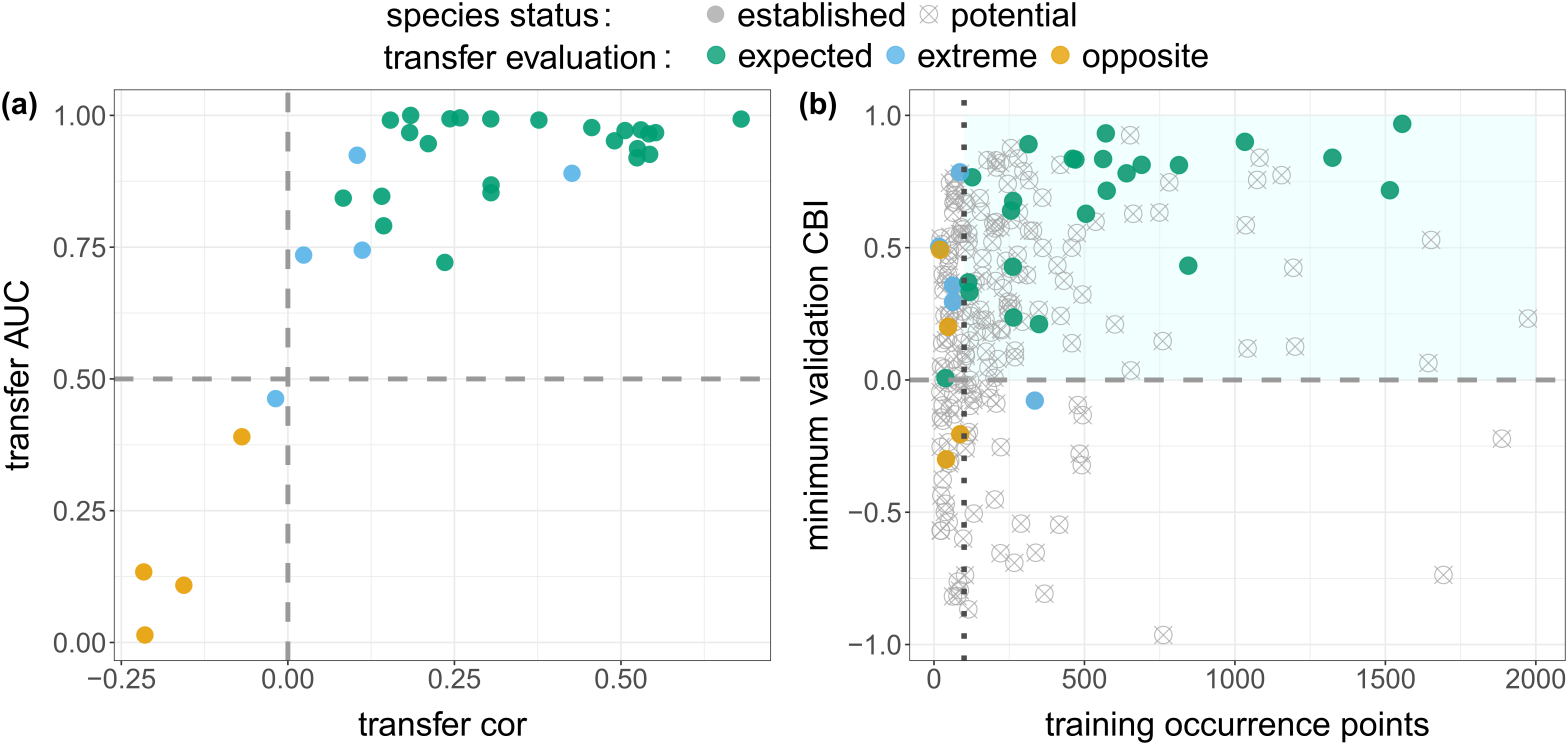
Selection of metrics associated with model transferability (step 2 of the workflow in Figure 1). We assessed which model evaluation metrics and values indicated low accuracy of model transfers for established species, and then used them as criteria for filtering transfers of unestablished species that lack the data to evaluate model transferability directly. (a) For established species, transfer metrics (cor [Pearson correlation] and AUC) allow for distinguishing “expected” transfers from “opposite” transfers (i.e., relatively lower suitability at occurrence points than elsewhere). (b) Input data (number of training points) and model‐validation metrics (minimum validation CBI across spatial cross‐validation partitions) as proxies for transferability: Models that fall in the light‐blue region are inferred to result in an “expected” transfer, while those outside are mainly “opposite” or “extreme” transfers (i.e., largely overpredicted or geographically bimodal and not matching occurrence points patterns). Established species (solid circle) are color‐coded by their transfer evaluation: Yellow corresponds to “expected” transfers, orange to “extreme” transfers, and red to “opposite” transfers. Unestablished species are represented as open circles in panel b. Only unestablished species within the blue‐shaded area of panel b were used for the rest of the workflow.

Although all established species with opposite transfers had transfer metric values that suggest a performance worse than random correspondence between the model and the independent test data (validation AUC < 0.5 and cor < 0; Figure 2a; Appendix S1: Sections S2 and S3, Figure S4), extreme transfers tended to have transfer metric values similar to those of the expected transfers (validation AUC > 0.70 and cor > 0.1; Appendix S1: Sections S2 and S3, Figure S4). Therefore, we searched for metrics describing input data and model validation that allowed us to clearly separate all dubious transfers from the rest of the predictions. We found that considering the number of occurrence points for the inhabited range (number of points used for model training; Figure 2b; Appendix S1: Section S4, Figure S5a) and the “minimum validation CBI” among spatial cross‐validation partitions (indicating the poorest performance for a given combination of model settings; Figure 2b; Appendix S1: Section S4, Figure S5b) allowed for this separation. Species with less than 100 occurrence points for model training and negative minimum CBI values for model validation were best associated with all dubious transfers (both extreme and opposite) for the established species (Figure 2b; Appendix S1: Figure S5c). Only 1 of 24 expected transfers (*Technomyrmex brunneus*) had values corresponding to those of a dubious transfer based on our criteria, which filtered out about 30% of established species (10 out of 33). This allowed us to infer dubious transfers for established species directly from the input data and model‐validation metrics, which is available information for unestablished species. We thus used these criteria to filter out 95 exotic species, which is 51% of those we had initially considered; the remaining 90 species were thus inferred to have expected transfers (Figure 2b).

### Potential alien species establishments and assemblages source regions

**FIGURE 3:**
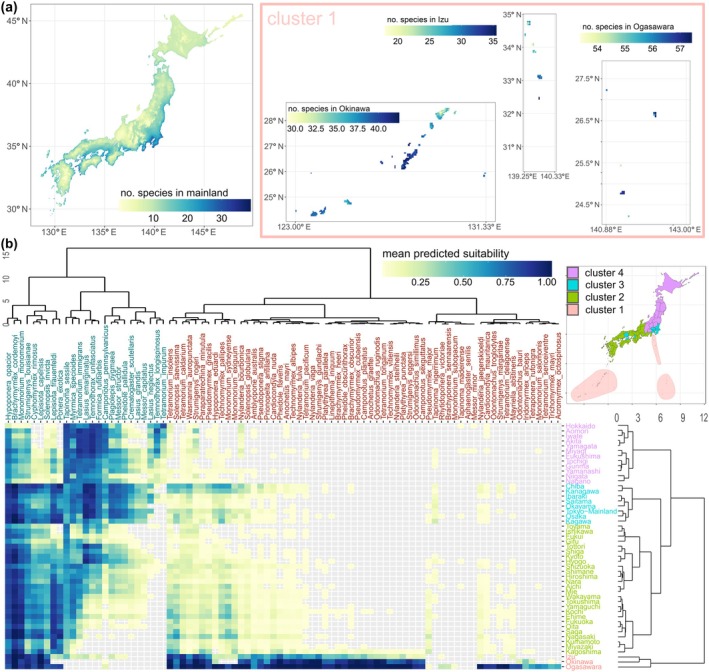
Richness and occupancy predictions clustered to define assemblages of threat species (step 3 of the workflow in Figure 1). For ants known to have been transferred to other areas by humans as alien species but are not yet present in Japan: (a) Estimated richness of these species calculated by summing continuous species distribution model predictions for mainland Japan and the smaller archipelagoes (cluster 1 in next panel). (b) Predicted occupancy of unestablished species by prefecture (mean suitability). Dendrograms show the clustering of species and prefectures estimated by hierarchical cluster analysis (Euclidean distance, Ward clustering), and colors for text and the inset map refer to the cluster assigned to each prefecture.

Of the 90 unestablished species that passed the filter, after masking areas below the 10‐percentile suitability value and those with negative MESS values, 86 species had predicted potential distribution in at least one prefecture of Japan (threat species). Across the country, the estimated richness calculated by summing these species' continuous predictions had a minimum of 0 and a maximum of 57.42, with a strong latitudinal effect where lower latitudes show higher predicted risk of establishment (Figure 3). We determined four clusters that best represented the different potential ant threat assemblages that could be newly established in Japan (Figure 3b, inset map). For the mainland islands of Japan (Kyushu, Shikoku, Honshu, and Hokkaido; clusters 2–4), we estimated that the coastal regions have a higher risk of establishment than inland regions with higher altitudes (Figure 3a). The three smaller archipelagoes (temperate Izu, and subtropical Okinawa and Ogasawara; cluster 1) had a higher risk of establishment than the rest of the country (Figure 3a). The prefecture‐level occupancy showed that Akita (in northeast Japan) had the lowest prediction (25 threat species) and Ogasawara had the highest (67 threat species; Figure 3b).

At the cluster level, we can observe that the climatic differences with increasing latitude result in lower predicted richness and mean suitability for most threat species (Figure 3b). The northernmost cluster (cluster 4), composed of prefectures above 35° N, had the lowest number of predicted threat species (58). Notable threat species for this cluster include the European species *Tetramorium immigrans*, which has been introduced and spread widely in North America, and the common North American species *Tapinoma sessile*. The predicted species assemblage for the southern archipelagoes (cluster 1, including 83 species) contains a long list of tropical and subtropical species, including damaging species such as the little fire ant (*Wasmannia auropunctata*), the raspberry crazy ant (*Nylanderia fulva*) and the red‐imported fire ant (*Solenopsis invicta*; Figure 3b). Clusters 2 and 3 (in the central part of the country) presented species assemblages shared with those extremes and had 78 and 65 predicted species, respectively, and these central clusters are also highly suitable for the red‐imported fire ant (Figure 3b).

**FIGURE 4:**
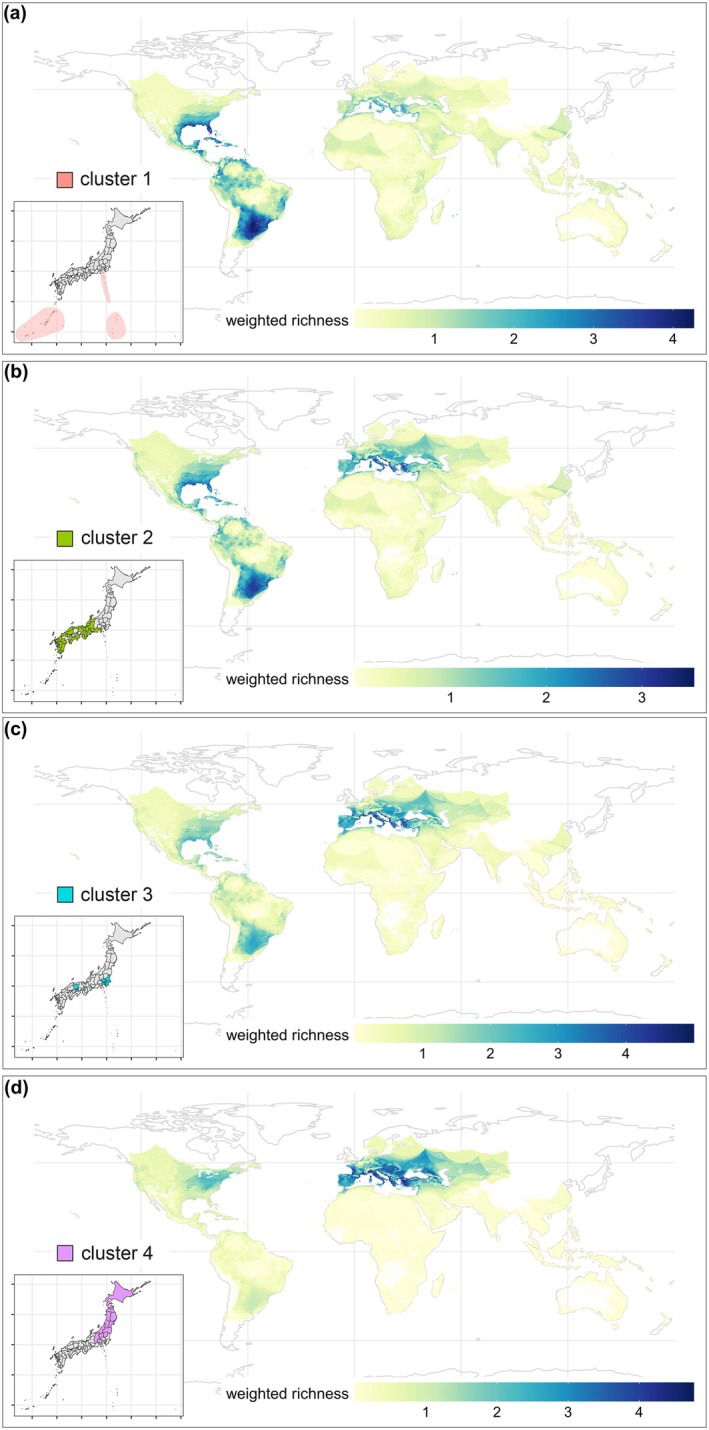
Source hotspots for the predicted threat assemblage of each cluster (step 3 of the workflow in Figure 1). The clusters are defined by the similarity across prefectures of the predicted alien ants with no history of invading Japan (shown in Figure 3). Weighted richness values represent the similarity between the assemblage of the cluster and the global region. (a) Cluster 1 includes the three smaller, southern archipelagoes: Okinawa prefecture, Ogasawara Islands, and Izu Islands. (b) Cluster 2 represents western Japan, including most of the prefectures in Kyushu, Shikoku, Chugoku, Kansai, and Chubu regions. (c) Cluster 3 is composed of some southeastern prefectures (Ibaraki, Chiba, Saitama, mainland Tokyo, Kanagawa) and several others in western Japan (Okayama, Osaka, and Kagawa). (d) Cluster 4 is the most northern cluster, including Niigata, Nagano, Gunma, Tochigi, and Yamanashi prefectures, as well as the Tohoku region and Hokkaido prefecture.

The predicted ant threat assemblage for the more southern archipelagoes (cluster 1) has two main source hotspots in subtropical regions of the Americas; however, there are also areas with high weighted richness of threat species in southern Europe as well as the Neotropics (Figure 4a). As mentioned before, the threat species with potential distributions in central Japan (clusters 2 and 3) share species with the northern and southern clusters and also share the same global hotspots (Figure 4b,c). Northern Japan (cluster 4) has one main estimated source hotspot in the temperate zones of Europe (Figure 4d). From the central clusters, the eastern cluster (cluster 3) is more similar to the north, while the western cluster (cluster 2) is more similar to the south. It is worth mentioning that potential sources of threat species for all clusters could be anywhere on the globe (except for extremely cold regions in the far north and south). The threat species are distributed across all continents, including Africa although to a lesser extent South Asia, Southeast Asia, and Oceania (Figure 4).

### Effect of trade on the predicted ecological patterns

**FIGURE 5:**
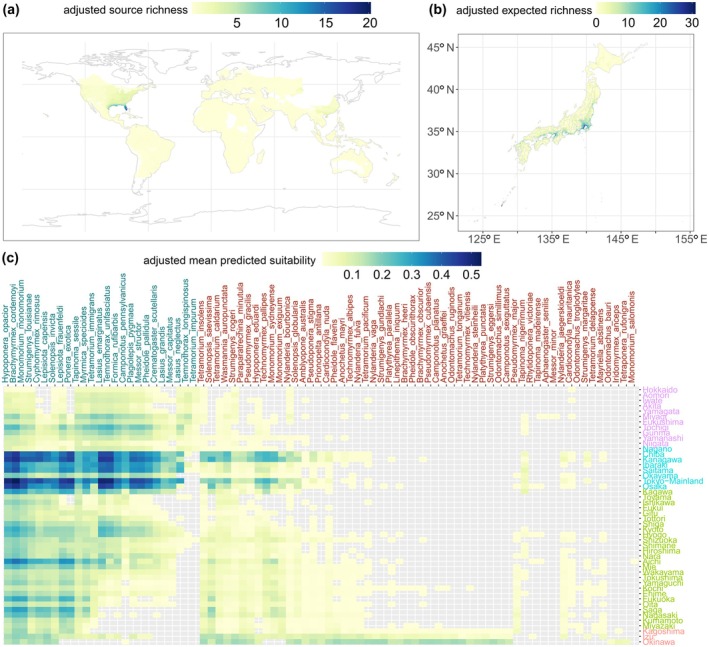
Source hotspots, weighted richness, and occupancy predictions for assemblages of threat species adjusted for introduction and spread risk. (a) Sources of unestablished threat species are adjusted by the trade volume of each country with Japan. (b) Weighted richness of unestablished threat species adjusted by an estimate of introduction and spread risk within Japan (combination of distance to major ports and human population density). (c) Occupancy of unestablished threat species per prefecture in Japan after adjustment in panel (b).

To incorporate introduction risk, we modified our predictions for the establishment and potential sources by combining estimates of human‐assisted dispersal of alien species (Appendix S1: Section S5) in two ways. First, the multiplication of source region weighted richness predictions with cargo volume reaching Japan per country (Appendix S1: Figure S6a) reduced the predicted risk of some countries that, ecologically, are potential hotspots for threat species (Figure 5a) yet do not have heavy trade with Japan—most of the largest trading partners were nations in the temperate zone. Second, the multiplication of the risk of establishment predictions for Japan with the estimates of the risk of spread within the country (Appendix S1: Figure S6b) resulted in a considerable shift in the areas at risk, with the highest values now closest to the main ports and the most densely populated areas of the country (mostly clusters 2 and 3), as well as an overall decrease in mean suitability of threat species over the whole country (Figure 5b). However, the total number of threat species did not change drastically, with 81 out of 86 species still predicted to establish and spread within the country (Figure 5c).

## DISCUSSION

The purpose of our study is to reveal potential threats of alien establishment in a new region and their known locations from a global pool of understudied species. As such, we sacrifice details of each species for a broad‐based analysis. Our analysis currently provides a macroscale horizon scan of potential future introductions of alien ants to Japan. Two of the five ant species included in the list of “100 worst invasive species” (the red‐imported fire ant, *S. invicta*, and the little fire ant, *W. auropunctata*; Lowe et al., 2000) are not yet established in Japan but our study predicts high suitability in some parts of the country, even after accounting for the risk of introduction and spread. However, our results also identify the potential emergence of “unexpected” alien species, for example, the tawny crazy ant (*Nylanderia fulva*), which is native to South America but has invaded the United States in recent decades and caused ecological and economic damage (Wang et al., 2016). Despite not being previously identified as a high‐threat species to Japan, the invaded areas in the United States also correspond to high‐risk potential sources to Japan in our analysis, even after adjusting for introduction risk. While species‐specific assessments are beyond the scope of this paper, by generating a list of potential threat species we take a first step toward a more comprehensive assessment of target species for which more detailed studies may follow.

The tawny crazy ant example above also emphasizes that to identify the measures that can be implemented to reduce the risks of the introduction and establishment of alien species, it is of critical importance to understand not only which alien species are likely to invade new regions, but also which areas of the globe are likely to be source areas of the entire ensemble of threat species. Current global trade patterns and volumes allow for the movement of a large number of species from anywhere in the world, including individuals from their native or alien ranges, that could pass unnoticed when active preventive measures are not in place (Costello et al., 2007). Examples abound but include well‐known cases of bark beetles (Liebhold & Brockerhoff, 2017), ants (McGlynn, 1999; Wong et al., 2023), and zebra mussels (Leung et al., 2002).

In this study, we examined which of the global pools of alien ants pose a threat to Japan based on climatic suitability and mapped the location of these threats. We found that while there is a risk of the establishment of new alien ants across the whole country, the smaller, southern archipelagoes of Japan are at higher risk. We also found that species assemblages predicted to be able to establish in northern and southern Japan are not only distinct but could potentially be imported from different regions of the world: temperate Europe and tropical and subtropical regions of the Americas, respectively. In comparison, the predicted species assemblages and sources for central Japan are a mixture of these two regions. Despite the compromises made for the SDMs, we were able to estimate the risk of establishment for the pool of candidate species, identify threat assemblages, and determine the location of predicted source areas for these assemblages around the globe. Importantly, this workflow can be used for other taxonomic groups of concern to directly inform alien species management and policy at the country level.

### Ecological realism and relevance of our predictions

SDMs are a critical but imperfect tool for future projections, with some limitations that need to be identified and understood (Araújo & Guisan, 2006; Lissovsky et al., 2021; Lissovsky & Dudov, 2021; Srivastava et al., 2019). While we followed recommended practices for variable selection (Jiménez‐Valverde et al., 2011; Phillips et al., 2017; Watari et al., 2021), data cleaning and optimization (Kass et al., 2022), algorithm selection (Duque‐Lazo et al., 2016; Kaky et al., 2020), and model parametrization and selection (Kass et al., 2022), we encountered previously identified difficulties associated with transferring SDMs to new environmental conditions (Morey & Venette, 2020; Petitpierre et al., 2017). Our approach to avoid potentially incorrect transfers was to evaluate model transfers for alien species that had known occurrences in Japan, derive criteria based on model‐validation metrics to infer which transfers are likely to be poor, and then apply these criteria to the alien species with no history of invasion (unestablished) in Japan to filter out dubious transfers as their model transfers could not be directly evaluated. When independent post‐release occurrence data is available for alien species, it could be used for direct assessments of model transferability to inform model selection (Sutton & Martin, 2022). Nonetheless, not all post‐release occurrence data may represent individuals originating from established alien populations, and thus may not be representative of the environments associated with the alien range at later stages. Additionally, such data is typically only available for intentionally introduced species whose true source of introduction (usually the native range) is known (Sutton & Martin, 2022), which limits its use when considering multiple species that often have limited information.

The strategy used for the unestablished species in this study involved removing dubious transfers from the downstream analysis to derive results with relatively higher confidence that could be applied to conservation measures. A sufficient number of occurrence points used to train the models for the unestablished species (minimum 100) was critical to obtain reliable predictions of potential establishments in the transfer region. Compounding issues of sample size and sampling biases (Peterson et al., 2011; Phillips et al., 2009), knowledge gaps for species distributions (Costa et al., 2010), and challenges in taxonomy (Pyšek et al., 2013) further limit our ability to derive highly reliable predictions of potential shifts in distribution for most insects. While we think our approach can derive reasonable patterns of potential new invasions and their current source hotspots, we recognize that removing species from an analysis based on inferences drawn from other species is not ideal. In this study, we excluded 96 potential new invaders (52% of the original exotic species pool) that had too few occurrence records or model‐validation metrics that did not meet our criteria for expected model transfers. These species could still represent threats to Japan and potentially have severe impacts, but their models were inferred to result in a dubious transfer. Moreover, there is a vast pool of species not yet known to be transported by humans which were not included in this analysis, thus we do not derive predictions for them but maybe potential future invaders (Seebens et al., 2018). Therefore, we consider that our current results are conservative and likely underestimate the total potential establishment of alien ants in Japan.

Transferability of SDMs is a heavily discussed topic (e.g., Morey & Venette, 2020; Sutton & Martin, 2022) that relies on many factors like variable selection (Jiménez‐Valverde et al., 2011; Lissovsky et al., 2021; Petitpierre et al., 2017), algorithm choice (Duque‐Lazo et al., 2016; Lissovsky & Dudov, 2021), and the viability of model assumptions (Richmond et al., 2010). One SDM assumption that is particularly difficult to meet when predicting global transfer is confirming that climatic conditions between the training and transfer regions are analogous, as these regions can differ considerably in climate and necessitate extrapolation. Additionally, even for models with good transferability, predictions of potential distributions in new areas might be inaccurate if the species undergoes a niche shift upon establishment (Bates et al., 2020; Battini et al., 2019; Macêdo et al., 2021). This is not an unrealistic scenario, as population growth and spread are shaped by multiple factors that could promote or limit their spread capacity in a given region. Some other known processes affecting the invasion process are competitive release (Tingley et al., 2014), plastic behaviors (Chapple et al., 2012; Sagata & Lester, 2009), and innate genetic or ecological advantages possessed by alien populations, which have been shown for ants (Eyer et al., 2018; Fournier et al., 2019; Tsutsui et al., 2000). All of these factors are not currently implementable in typical SDM workflows and the development of new modeling methods would be needed to include them. Moreover, data availability for understudied taxa at both regional and global scales remains a limitation. However, should data in these realms expand in the future, integrating these factors into SDM analyses would be a promising innovation.

### Implications and applications for alien species management

With our modeling approach, we predicted that different assemblages of species threaten establishment among the different administrative regions of Japan due to strong differences in climate and that those species can potentially invade from different regions of the world. Our results show that the smaller, primarily southern archipelagoes of Japan (Ogasawara, Okinawa, and Izu) are at higher risk of establishment compared to the rest of the country, both because we predicted more threat species there, and because the mean estimated suitability on these islands is higher than on the mainland for most threat species. However, this could be due to the relatively small area of the archipelagoes where most areas are highly suitable for the threat species, compared to the mainland where areas of high and low suitability occur within the same prefecture and are averaged together. The Japanese government already spends more money per unit area to mitigate established alien species in the archipelagoes than in the mainland (Watari et al., 2021). This is likely a good management strategy, as the effects of alien species on small islands are relatively stronger and island isolation does not confer protection (Moser et al., 2018). Nevertheless, current management programs in Japan are heavily focused on the protection of human livelihood (Watari et al., 2021), but as some alien species might not represent direct threats to humans, they may be overlooked. A broader approach to potential new introductions and establishments which considers other threats such as habitat degradation, agricultural damage, or other consequences that can indirectly harm human society (Siddiqui et al., 2021) is likely required. Other island nations likely face similar challenges in protecting their environment and population from potentially harmful species (Reaser et al., 2007; Xu et al., 2022).

This study identifies alien species with potential climatic suitability in Japan and the global regions in which they are currently found, and it also estimates introduction and spread risks based on overall commodity imports to Japan and human population density within Japan. However, the introduction is a complex process, and predictions could be improved further with a more focused analysis. First, a next step would be to integrate these threats with explicit networks of global shipping, during which the whole transit pathway should be considered as well as the trading connections to countries considered dispersal hubs for alien species (Capinha et al., 2023). Therefore, partnerships between trading countries and those with intermediate trading relationships (Gippet et al., 2019; Xu et al., 2022) are needed to implement effective preventive measures. Furthermore, not all types of cargo would be equally likely to introduce any given group of species; for example, trade in automobiles may be less risky than agricultural or horticultural products, and high‐risk cargo may also vary across species—this could be determined and integrated as well in future work.

Our work also helps to bridge prevention (at departure; sensu Blackburn et al., 2011) and containment (at arrival; sensu Blackburn et al., 2011) with a practical workflow that identifies, according to cargo destination, the sites where prevention should be implemented. Containment methods at the arrival country primarily rely on the detection (i.e., finding and correctly identifying) of all potential alien species able to establish in the country, yet there may not be enough information and trained staff available to make proper decisions (Pyšek et al., 2013). The use of DNA screening methods (Darling & Blum, 2007), automatic visual recognition (Demertzis & Iliadis, 2017), or systematic treatment to eradicate target organisms (Hallman, 2007) could be good practical alternatives that alleviate the demands for on‐site experts and possible excessive infrastructure at each entry point. Nonetheless, preventive measures prior to departure might be even easier to implement by ensuring clean cargo.

Our study on ants that present threats to Japan constitutes a test case that is of intrinsic interest, but more broadly establishes a workflow that can be applied to other regions and taxa. Ants include some of the most detrimental and costly invasive species in the world (Angulo et al., 2022; Lowe et al., 2000; Xu et al., 2022). Additionally, Japan is a large island nation with broad latitudinal coverage, allowing us to investigate how predicted alien threat species assemblages and source regions differ by climate and if remote islands are at higher risk. Nonetheless, this analysis is easily extendable to any other taxon with ample occurrence data to train SDMs that have good transferability (in this study, these required at least 100 occurrence records). For this study, we used species occurrence data from the GABI database, a data resource specializing in ants (Guénard et al., 2017), but other biodiversity databases can be used for other taxa, such as the Global Biodiversity Information Facility (GBIF, 2023) or iNaturalist (iNaturalist, 2023), as long as data on native and alien records are available and reliable. In all cases, however, appropriate data cleaning and curation are advised. Further, although we focused on a single nation, this workflow can also be applied to other extents of different sizes, including continental regions, as the environmental and geographic data we used for this study are freely available for the globe. Nevertheless, it is important to note that SDM transferability is dependent on the quality of the input data as much as the model performance; therefore, it is advised to carefully study the transferability of the models for each region (e.g., using previously established species as in this study) before drawing conclusions.

This study predicted the country‐specific potential of establishment for a whole insect family of global concern and potential source regions around the world. While species distribution modeling based on occurrences is far from perfect given the complexity of invasion ecology, in the face of an ongoing biodiversity crisis, we believe this is a reasonable approach until more data and knowledge are available for alien species. In particular, we recommend the use of other information types whenever available, such as genetic data (Boissin et al., 2012; Lovrenčić et al., 2022) or information on species interactions (Lany et al., 2017) as these would likely further increase the accuracy and realism of predictions. Moreover, such macroscale approaches need to work synergistically with more focused species‐specific assessments. If modeling approaches such as these can be used to guide the deployment of resources more efficiently, they can be a significant contribution to efforts to limit the negative impacts of alien species.

## AUTHOR CONTRIBUTIONS

Evan P. Economo conceived the idea. Evan P. Economo, Jamie M. Kass, and Yazmín Zurápiti designed the methods. Jamie M. Kass downloaded and processed the GABI data. Yazmín Zurápiti performed the data cleaning, assembled the workflow with Jamie M. Kass, and performed the data analysis and interpretation. Yazmín Zurápiti drafted the manuscript. Benoit Guénard, Evan P. Economo, Jamie M. Kass, and Yazmín Zurápiti revised the manuscript and approved the final version for publication.

## CONFLICT OF INTEREST STATEMENT

The authors declare no conflicts of interest.

## Supporting information


Appendix S1.

## Data Availability

Data and code (Zurápiti et al., 2025) are available in Zenodo at https://doi.org/10.5281/zenodo.15541531. Other data (WorldClim v2 rasters and Natural Earth vectors) are publicly available with those items cited within the text and code.
