# Supplementary material for: Forecasting alien species establishment and source regions: Quantitative assessment of potential ant invasions in Japan
Source: Ecol Appl. 2025 Jul 14;35(5):e70071. doi: 10.1002/eap.70071 (PMC12257433; doi:10.1002/eap.70071)
Supplement: Supplementary file 1 — Appendix S1. [file EAP-35-e70071-s001.pdf]

List of authors:

Yazmín Zurápiti, Jamie M. Kass, Benoit Guénard, Evan P. Economo

Manuscript title:

Forecasting alien species establishment and source regions: Quantitative assessment of potential ant invasions in Japan

Journal name:

Ecological Applications

Appendix S1: Reasoning behind the selection of transfer and training metrics as a proxy of transferability.

Alien species are predicted to continue spreading and accumulate at continental scales, so to inform control measures, there is a pressing need for better predictions of invasion risk (Cuthbert et al. 2022; Leung et al. 2002). Species distribution models (SDMs) have become a valuable tool to predict the ranges of alien species and their potential spread across new regions (Escobar et al. 2014; Jiménez-Valverde et al. 2011; Srivastava, Lafond, and Griess 2019). Such predictions may extrapolate beyond the training data of the model, and it is still unresolved how best to evaluate their accuracy and ecological realism (transferability). One possible solution for species that are already established in an area of interest is the use of independent post-release data (Sutton and Martin 2022). However, such data is not available for alien species yet unestablished in the area. Moreover, the main objective is to prevent their introduction and potential establishment.

Therefore, in this study we propose to infer the SDM transferability of unestablished species by determining the accuracy and realism for the established species' models, then developing transferability criteria based on the latter (step 2 of the workflow, Figure 1 of main text).

Section S1: Suitability prediction patterns that may arise from SDM transfers

To evaluate transfer performance of SDMs for species already established in Japan, we categorized the realism of their predictions based on the species' ecology and calculated different model performance metrics (next sections) that determine model accuracy to predict the alien records, which correspond to new data (i.e., not included in model training data). The prediction categories were: "expected" when the predicted suitability patterns were generally high where the occurrence records are found or in areas with similar climate, and generally lower elsewhere (Figure S1), or "dubious" when these patterns were unexpected based on the species' known biology. The dubious category was further broken down into one of either "opposite" (predicted patterns that had relatively lower suitability at the occurrence points than elsewhere; Figure S2) or "extreme" (predicted patterns were overpredicted or had geographically bimodal suitability predictions that did not match the occurrence points; Figure S3).

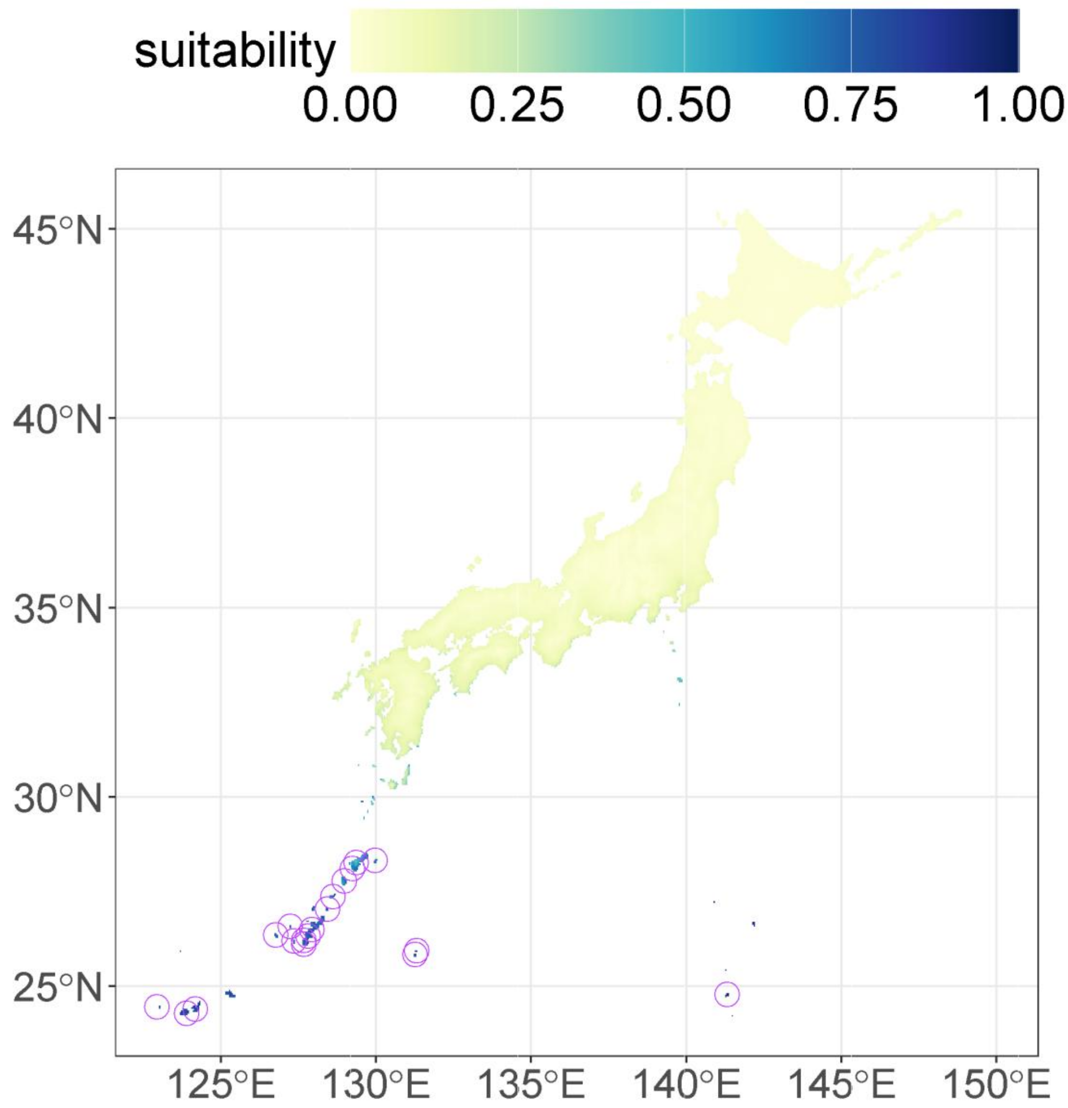

Figure S1: Example of a model transfer for *Anoplolepis gracilipes* with expected suitability patterns for alien species already established in Japan. We refer to these transfers as "expected" because the predicted suitability patterns matched those of their known occurrence points in Japan (purple open circles). In this analysis, there were 24 species with expected patterns. Suitability predictions for Japan were made with models trained on the species' global range outside Japan. The purple open circles were used to assess the accuracy and realism of model transfers.

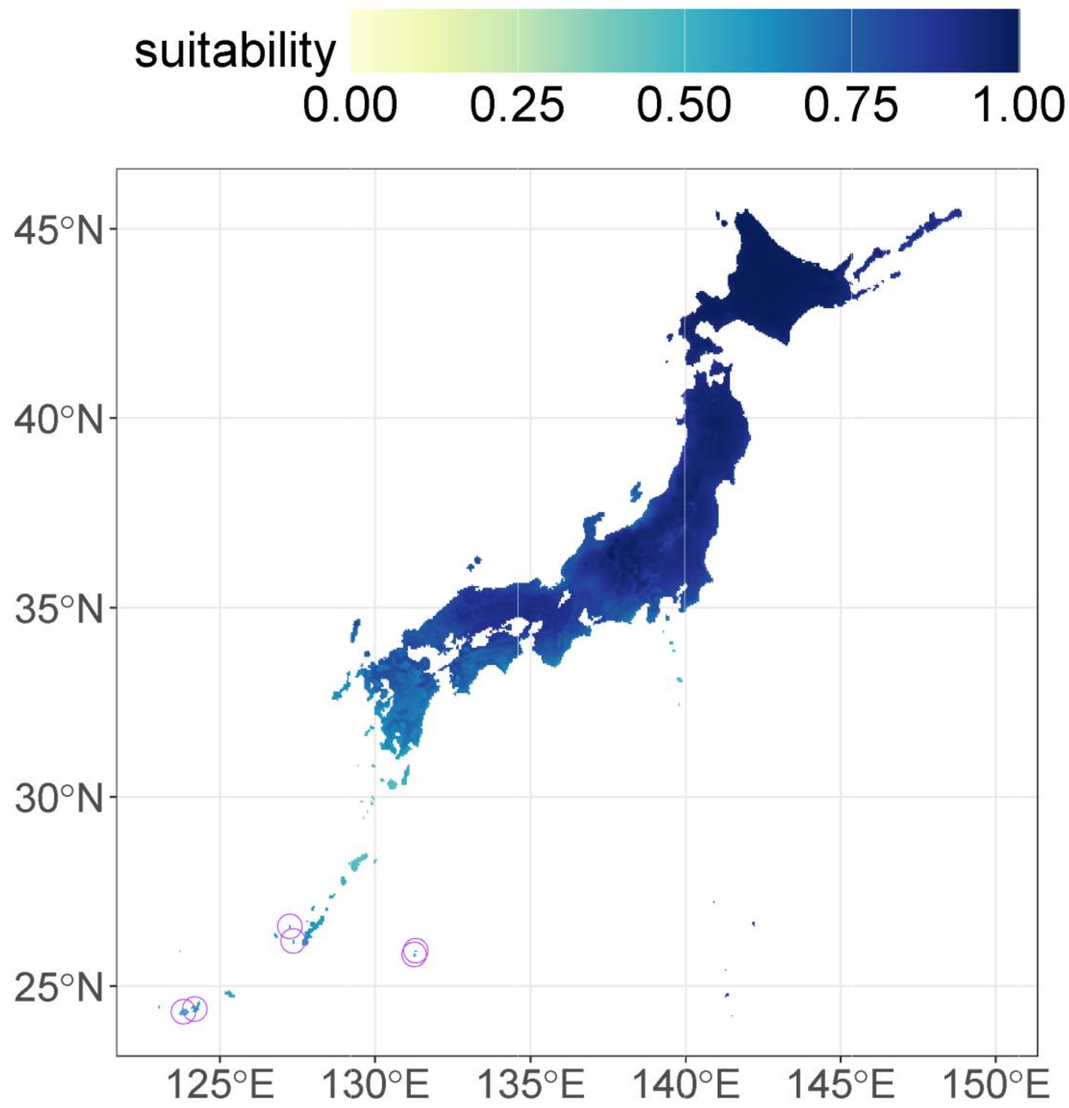

Figure S2. Example of a model transfer for *Tetramorium smithi* with opposite suitability patterns for alien species already established in Japan. We refer to these transfers as "opposite" because the predicted suitability patterns showed relatively lower suitability at the species' occurrence points (purple open circles) than areas where we lack occurrence data for the species. In this analysis, there were 4 species with opposite patterns. Suitability predictions for Japan were made with models trained on the species' global range outside Japan. The purple open circles were used to assess the accuracy and realism of model transfers.

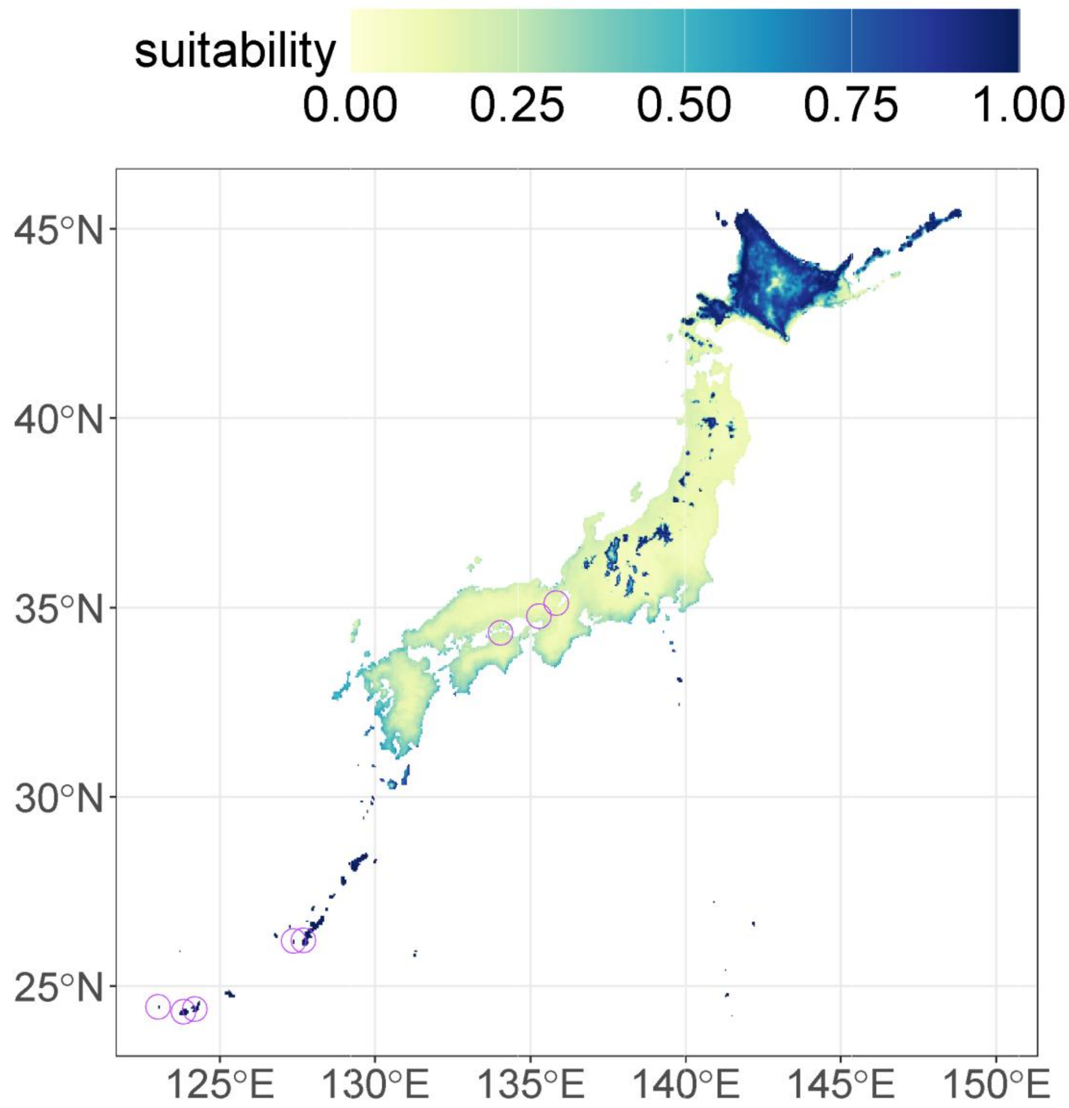

Figure S3. Example of a model transfer for *Hypoponera ragusai* with extreme suitability patterns for alien species already established in Japan. We refer to these transfers as “extreme” because the predicted suitability patterns appeared overpredicted or geographically bimodal and did not match the species occurrence points (purple open circles). In this analysis, there were 5 species with extreme patterns. Suitability predictions were made with models trained on the species’ global range outside Japan. The purple open circles were used to assess the accuracy and realism of model transfers.

Section S2: Explanations of transfer performance metrics

The metrics we chose to determine model accuracy were the Continuous Boyce Index (CBI), Pearson correlation coefficient (cor), and the Area Under the Curve (AUC) of the receiver operating characteristic (ROC). CBI is calculated using presence data only, and thus is advocated for use with presence-background SDMs that do not employ absence data (Hirzel et al. 2006, di Cola et al. 2017). CBI estimates how consistent model predictions are with the spatial distribution of the presence data, and we used the R package *ecospat* to calculate it (di Cola et al. 2017). Values range from -1 to 1, where values closer to 1 represent increasingly consistent predictions, zero represents random predictions, and values closer to -1 represent inconsistent predictions (i.e., lower suitability for presences than absences or background data). The other two metrics use both presence and absence data, where background data is substituted for absence in the case of presence-background SDMs. The "cor" metric is the point biserial Pearson correlation coefficient calculated on the model predictions for presences and absences (or background data) and range from perfect correlation (1) to no correlation (0; Elith and Graham 2009). AUC measures the ability of the model to discriminate between presences and absences over a continuous range of thresholds (Fielding & Bell 1997). This metric ranges from 0 to 1, where values closer to 1 represent better discrimination, 0.5 represents random discrimination (but see Lobo et al. (2008) for differences in interpretation for presence-background SDMs), and values closer to 0 represent discrimination ability that is worse than random (i.e., predicting absences higher than presences). Both metrics were calculated with the evaluate function in the R package dismo (Hijmans 2023).

Section S3: Relationships between transfer performance metrics

We compared the chosen metrics to understand their pairwise relationships and how they relate to our qualitative descriptions of the SDM predictions (Figure S4). We determined that CBI was not a good proxy to distinguish the quality of the transfers in our study because: 1) three species resulted in NA values yet had transfers that matched our expectations and thus should have positive values, and 2) although opposite and extreme transfer species had CBI values below 0, so did two other species with expected transfers. Therefore, it was not clear how to choose a value that could separate the species with expected transfers from the extreme and opposite ones. For example, CBI >= -0.52 includes all species with expected transfers, yet values below zero theoretically represent predictions worse than random, and this threshold also includes multiple transfers we identified as dubious (Figures S4a, S4b). On the other hand, expected transfers could be better bounded by both cor and AUC metrics with values above their random expectations (cor >= 0.07 or AUC >= 0.71; Figure S4c), though some extreme transfers were still within these bounding values (Figure S4c). Thorough investigations into why some models with low CBI values also had high AUC values or why some CBI values resulted in NA were outside the scope of this study, but rigorous comparisons are needed to aid interpretation when comparing these two metrics, which are frequently but not always correlated.

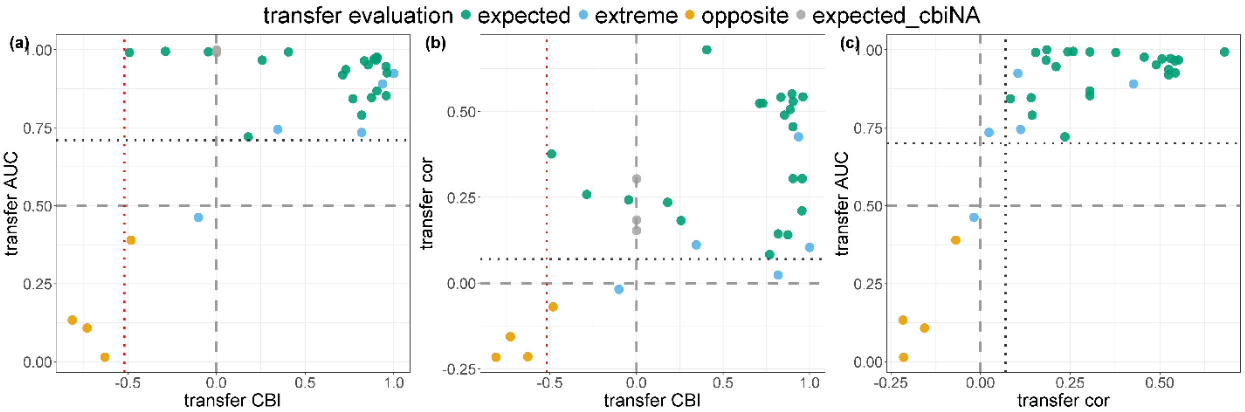

Figure S4: Evaluation of model transfer accuracy. Colors represent the transfer category: “expected” given the data, “opposite” to the data, or “extreme” patterns not expected from the data. Both “opposite” and “extreme” are subsets of the category “dubious” we use in this study. The category “expected_cbiNA” shows the three species for which the calculation of CBI failed (resulting in NA); a value of zero was used exclusively for visualization purposes. Light-gray dashed lines show the theoretical random values for the metrics. Dotted lines show the minimum values that bounded expected transfers — these lines are gray when the value is above the random expectation and red when the value is below. a) CBI vs AUC, b) CBI vs cor, c) cor vs AUC.

Section S4: Final selection of proxies for transferability

We explored a wide range of relationships between the transfer AUC, the raw data, and the model training metrics. However, we found that the total number of occurrence points used for modeling and the minimum validation CBI value (the lowest CBI value among all data partitions for spatial cross-validation; Kass et al. 2021) were the best and most consistent indicators of low model transferability (Figure S5). A low number of occurrence points (less than 100; Figure S5a) and a negative value of validation CBI in any given partition were good indicators of unexpected model transfers (Figure S5b). This aligns with expectations because if the model could not predict a withheld partition accurately during spatial cross-validation, this means it likely cannot transfer well to other regions. In the case of CBI, we can interpret that if any given partition performs worse than the random expectation (CBI <= 0); then the transfer will be dubious. Thus, our criteria require a species to have a minimum of 100 training occurrence points and positive CBI across all cross-validation partitions to ensure good transferability (Figure S5c).

To derive the community-level richness and occupancy predictions and their potential sources for the unestablished alien ants, we only included species with metrics that met the transferability criteria (Figure S5c). There is a possibility that a model can still transfer well despite being below the stated thresholds. However, we decided to include only the models in which we have the highest confidence to assure reliable predictions. This resulted in 90 unestablished species (48.6% of the data set) with high-confidence transfers and 95 unestablished species (51.4%) that were outside the inferred criteria (Figure 3).

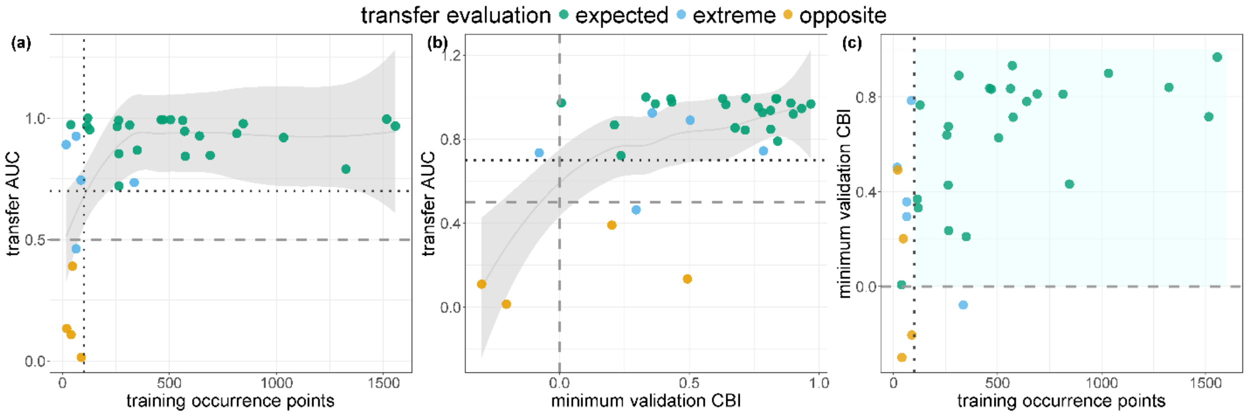

Figure S5: Selected training metrics for the transferability criteria and their relationships with different model transfers. a) Expected transfers have more than100 occurrence points for model training and a high transfer AUC, b) a minimum validation CBI value higher than 0 and high transfer AUC, and c) proxies for transferability derived from the established: the criteria require at least100 occurrence points for model training and a minimum validation CBI value higher than 0. Considering these metrics together clearly differentiates expected transfers from dubious predicted suitability patterns. Colors show the transfer category: "expected" given the data, "opposite" to the data or "extreme" patterns not expected from the data. Light-gray lines show the lower transfer AUC values that bound expected transfers determined in the previous section. Dark gray dotted lines show the theoretical random values of the metrics. The chosen transferability criteria used in this study (shaded in blue in panel c) correspond to a minimum of 100 occurrence points to train the model with and non-negative minimum validation CBI, which we used to infer the transferability of the unestablished species.

Section S5: Deriving human-assisted dispersal of alien species

To incorporate risk of introduction and spread into our estimates of establishment risk for unestablished alien species, we made two new maps. The first map is for introduction risk, which we estimated as the volume of cargo sent to Japan for all countries in the world. For this, we downloaded data on global trade to Japan from the UN Comtrade Database (https://comtradeplus.un.org) from 2015-2023, retaining only imports to Japan by sea, and derived the average value of trade goods per country in USD. We then standardized values to between 0 and 1, but did not transform the data otherwise to preserve the wide discrepancies in trade values among countries (Figure S6a). The second map is for risk of spread, which we estimated as the product of the inverse distance from “major ports” and the logarithm of human population density. In this case, we downloaded the datasets from Japan’s Digital Land Information download sites (port cargo information: https://nlftp.mlit.go.jp/ksj/gml/datalist/KsjTmplt-C02-v3_2.html, population density information: https://nlftp.mlit.go.jp/ksj/gmlold/datalist/gmlold_KsjTmplt-suikei140704.html) from the National Land Information Division. For the port cargo information, which is from 2023, we removed all ports with NA values for import tons, and designated the remaining ones as “major ports”. We performed an inverse distance weighted interpolation on Japan using a raster with the same 10 arc minutes resolution as the SDMs using the interpIDW function in the *terra* package (Hijmans et al. 2023) with the “field” argument set to the log of the import tons, “ radius” set to 5, “power” set to 1, and other arguments left at their default values. The human population density data for Japan corresponds to the "Heisei 22 Census" (year 2014). We log-transformed the values and rasterized the shapefile to match the resolution of the distance interpolation, then

multiplied the two rasters together and standardized the product between 0 and 1 to make an estimate of risk of spread within Japan (Figure S6b).

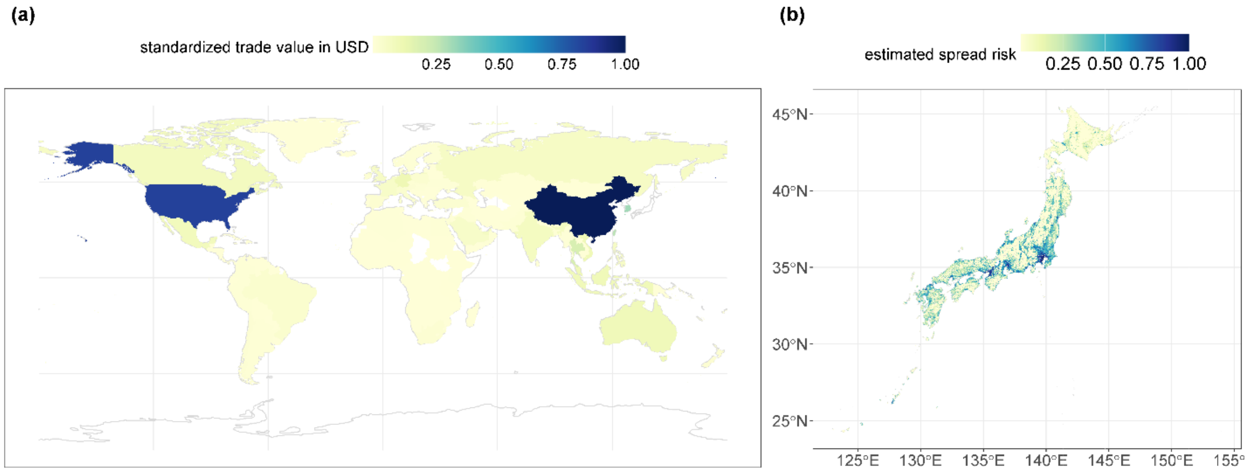

Figure S6: Estimates of introduction and spread risk based on human activity. a) Estimated introduction risk based on import value by export country, b) estimate of spread risk based on the multiplication of the distance to ports weighted by the trade value size of the port and the logarithm of the population density in Japan.

References

Cuthbert, R. N., C. Diagne, P. J. Haubrock, A. J. Turbelin, and F. Courchamp. 2022. "Are the '100 of the World's Worst' Invasive Species Also the Costliest?" Biological Invasions 24 (7): 1895–1904.

Elith, Jane, and Catherine H Graham. 2009. "Do They? How Do They? WHY Do They Differ? On Finding Reasons for Differing Performances of Species Distribution Models." Ecography 32 (1): 66–77.

Escobar, L. E., A. Lira-Noriega, G. Medina-Vogel, and A. T. Peterson. 2014. “Potential for Spread of the White-Nose Fungus (Pseudogymnoascus Destructans) in the Americas: Use of Maxent and NicheA to Assure Strict Model Transference.” Geospatial Health 9 (1): 221–29.

Hijmans, Robert J, R Bivand, K Dyba, E Pebesma, and MD Sumner. 2023. “Terra [R Package].” R Programming Language.

Jiménez-Valverde, A., A. T. Peterson, J. Soberón, J. M. Overton, P. Aragón, and J. M. Lobo. 2011. “Use of Niche Models in Invasive Species Risk Assessments.” Biological Invasions 13:2785–97.

Kass, J. M., R. Muscarella, P. J. Galante, C. L. Bohl, G. E. Pinilla-Buitrago, R. A. Boria, M. Soley-Guardia, and R. P. Anderson. 2021. “ENMeval 2.0: Redesigned for Customizable and Reproducible Modeling of Species’ Niches and Distributions.” Methods in Ecology and Evolution 12 (9): 1602–8. https://doi.org/10.1111/2041-210X.13628.

Leung, B., D. M. Lodge, D. Finnoff, J. F. Shogren, M. A. Lewis, and G. Lamberti. 2002. “An Ounce of Prevention or a Pound of Cure: Bioeconomic Risk Analysis of Invasive Species.” Proceedings of the Royal Society of London. Series B: Biological Sciences 269 (1508): 2407–13.

Lobo, J. M., A. Jiménez-Valverde, and R. Real. 2008. “AUC: A Misleading Measure of the Performance of Predictive Distribution Models.” Global Ecology and Biogeography 17 (2): 145–51. https://doi.org/10.1111/j.1466-8238.2007.00358.x.

Srivastava, V., V. Lafond, and V. C. Griess. 2019. “Species Distribution Models (SDM): Applications, Benefits and Challenges in Invasive Species Management.” CABI Reviews 2019:1–13.

Sutton, G. F., and G. D. Martin. 2022. “Testing MaxEnt Model Performance in a Novel Geographic Region Using an Intentionally Introduced Insect.” Ecological Modelling 473:110139. https://doi.org/10.1016/j.ecolmodel.2022.110139.
